# Minimum standards and development perspectives for the use of simulated patients – a position paper of the committee for simulated patients of the German Association for Medical Education

**DOI:** 10.3205/zma001239

**Published:** 2019-05-16

**Authors:** Tim Peters, Michael Sommer, Angelika Hiroko Fritz, Angelika Kursch, Christian Thrien

**Affiliations:** 1 hsg Bochum, Department für Pflegewissenschaft, Bochum, Germany; 2 TU Dresden, Med. Fakultät Carl Gustav Carus, Referat Lehre, Med. Interprof. Trainingszentrum (MITZ), Dresden, Germany; 3 University of Duisburg-Essen, Faculty of Medicine, Simulation Patient Program, Essen, Germany; 4 Medizinische Hochschule Hannover, Hannover, Germany; 5 Universität zu Köln, Köln, Germany

## 1. Foreword

Simulated patients (SPs) today are an integral and indispensable component of initial, further and continuing education in the health care system of German-speaking countries. SPs are (amateur) actors who are trained and take on the role of patients in teaching contexts in order to facilitate credible practice, examination and feedback scenarios. There are now several publications such as the AMEE Guide No. 42 “The use of simulated patients in medical education” [1] or the “Standards of Best Practice” [2], which adequately describe the current state of research and formulate a series of quality requirements. However, these recommendations and standards often refer to the use of SPs in the North American setting. This sometimes differs noticeably from European or German-speaking countries. In German-speaking countries, for example, the focus is much more on teaching than on examinations.

This position paper was prepared by the Committee for Simulated Patients^1^ of the German Association for Medical Education (GMA) in open forums with the participation of further SP experts from German-speaking countries in a multi-level consensual process.

The paper intends to supplement international developments with a German-language perspective which takes account of local circumstances. To this end, the SP method, the research situation and international criteria and standards for the use of SP are briefly outlined and a survey on the current status of the use of SPs is made. Building on this, minimum standards and development perspectives for German-speaking countries are formulated based on current conditions and the criteria called for in the international literature

This position paper was adopted on 09.19.2018 by the Committee for Simulated Patients at the annual meeting of the Society for Medical Education 2018 in Vienna. We would like to thank both the committee and everyone else involved in the process^2^. 

## 2. Background

### 2.1. Research status

The “SP” method was developed in the sixties under the term “programmed patient” by the American neurologist Howard Barrows to examine medical students in the field of typical neurological syndromes [3]. After initial teething problems and once resistance from the scientific community had been overcome [4], [5], the use of SPs became standard in exam contexts in North America [6], [7]. In the German-speaking world, their history has not yet been scientifically studied in broad terms. They were first used intermittently in the 1980s and then systematically from around the year 2000 onwards. Institutions pioneering SPs in their new model or reform study programs include the private university Witten-Herdecke and the Charité in Berlin, [8], [9], [10] and the entire spectrum of health professions in Switzerland. 

The terminology regarding simulated patients was at times inconsistent, both internationally and in German [1], [2], [11]. By now, however, terminology has been established which distinguishes the terms according to the field of application, as is customary internationally [7]. As a generic, the term simulated patient is used, which describes their use in teaching settings and is abbreviated to “SP”. In exam settings with a high degree of standardization, the terms “standardized patient” or “standardized SPs” [1] are used. The term “patient actor” is avoided because it is considered too trivializing. SPs also simulate other roles in health care, such as relatives or colleagues. However, these are exceptions, which is why people in general continue to speak of simulated patients, although more general terminology such as “human simulation” has already been established internationally.

For teaching, the use of SPs has various advantages [1], [5], [6], [12], [13], [14], [15], [16], [17], [18]:

- A variety of patient contacts and the presentation of relevant content or illnesses can be firmly scheduled in teaching.
- The use of SPs serves patient protection and is ethically justifiable. Thus, SPs can be used in teaching situations where working with real patients would be inappropriate (for example delivering bad news, embarrassing topics, conflict situations).
- SPs can repeatedly simulate certain personalities or illnesses. Both multiple contacts with different students and repetitions for individual students are equally possible.
- SPs can be trained for many disorders and desired behaviors (such as emotional responses, perceived pain).
- SPs can adjust the “difficulty” of the presentation to the desired level of challenge or learning objectives.
- The use of SPs in teaching is more effective in learning consultation-related skills than conventional teaching formats such as lectures or seminars.
- SPs allow learning in a safe environment.
- SPs can provide qualified feedback from the perspective of patients.
- The presentation of SPs can be standardized, thus creating comparable situations for learners and exam candidates. In addition, with SPs reliable and valid exams are possible.
- SPs can be available on demand. Their use is practical and comparatively cost-efficient.
- SPs in medical education are accepted and appreciated by both students and physicians.

Studies involving covert SPs have also shown that detection rates can be very low [19], [20], [21], suggesting that the presentation of SPs can be very close to real patients. 

#### 2.2. International criteria and standards

In order to facilitate the use of the SPs in teaching, examination and research, these are selected in advance and trained in terms of presentation, feedback and other criteria, if appropriate. Even if SPs ideally may be indistinguishable from real patients [22], the goal is less to create a totally realistic scenario [23] but rather to provide a credible scenario that allows for hands-on learning and examination [24]. There are two main publications in the international literature on corresponding quality criteria for the use of SPs. Although the AMEE Guide No. 42 by Cleland, Abe and Rethans [1] mainly describes the current state of research, it also implicitly and explicitly formulates a series of standards or quality criteria: 

##### 1. Clear selection criteria 

Amongst other things, SPs should be able to remember the relevant medical facts and background to their scripted role, adequately present the role, provide feedback and last but not least, work together with the other SPs and the team. They should bring a suitable attitude to the role and be reliable. Aspects such as age suitable for the role are also mentioned.

##### 2. A structured recruitment and selection process.

Here, the authors propose a multi-stage process that begins with a screening and an interview phase. Candidates receive information about the program and sit in on a simulation. Finally, a longer testing phase of SP tasks begins before the new candidates are accepted into the program. Differences between amateur actors and professional actors as well as the advantages and disadvantages of paying for SPs are addressed but no clear line is taken.

##### 3. Training for different types of assignments.

By way of example, various possible assignment scenarios in teaching and exam contexts are addressed and it is emphasized in advance that specially adapted training for the SPs for each scenario are of great importance.

The authors of the Standards of Best Practice (SOBP) published by the Association of Standardized Patient Educators (ASPE) published in 2017 go one step further [2]. As a result of an international consensus process, they set a range of specific standards for the use of SPs and categorize them into various subject areas. In the following, the five top categories or domains and the underlying principles are listed in the English original. For reasons of clarity, the enumeration of the individual sub-points or practices is dispensed with; please refer to the publication itself for these.

1. Safe work environment

- Safe work practices
- Confidentiality
- Respect

2. Case development

- Preparation
- Case components

3. Training SPs

- Preparation for training
- Training for role portrayal
- Training for feedback
- Training for completion of assessment instruments
- Reflection on the training process

4. Program management

- Purpose
- Expertise
- Policies and procedures
- Records management
- Team management
- Quality management

5. Professional development

- Career development
- Scholarship
- Leadership

The minimum standards formulated by the Committee for Simulated Patients should also align themselves with these existing standards and criteria for high-quality use of SPs. In addition, however, the established “SP traditions” of German-speaking countries as well as the predominant institutional, scientific and working cultures are taken into account.

#### 2.3. Current status in German-speaking countries

For reasons of clarity, reference is made here only briefly to the published survey on the current status of SP programs in German-speaking countries [25]. This survey was conducted in 2016 on the initiative of the GMA Committee on Simulated Patients in Germany, Austria and Switzerland and subsequently evaluated and published. The returns of 38 institutions about size, structure, function and goals of the respective programs formed an essential basis for the discussions on the position paper. 

Without going into detail on individual aspects, it became apparent that the use of simulated patients is now an established didactic method in German-speaking countries, widely used in medical teaching (1,290 SP hours per year on average for all answers) but that the actual implementation in the faculties is also very heterogeneous, also with regard to different traditional practices in each country. Differences in day to day practice are noted both for organizational aspects such as location within the institution or financing, in the qualification of the SP coaches, established work processes (such as recruitment or training) as well as didactic orientation (for example actual areas of use in medical education or feedback instruments used). At the same time, general and inclusive overarching principles and structures have emerged. 

#### 2.4. Structure and orientation of the development workshops

Several workshops, which over a period of four years accompanied the process of creating the position paper, acted as central interfaces and coordination platforms. The workshops took place every six months at the GMA annual meetings and the skills-lab symposiums. On average, they had 10-15 participants made up of SP program managers or SP trainers, who were usually also members of the GMA Simulated Patients’ committee. Work was initially carried out in three working sub-groups (literature review, survey or current status, minimum standards), which presented their results at regular intervals. The discussed consensus was subsequently returned to the groups for further processing. From the Skills-Lab Symposium 2017 onwards, the results were gradually brought together and discussed in detail in the larger group. The authors of the position paper incorporated the discussion results into the current draft between workshops, which in turn served as a basis for discussion for the next workshop.

The following overview clarifies orientation of the content in the individual workshops and at the same time is to be regarded as the timeline of the preparation process (see table 1 (Tab. 1)).

## 3. Minimum standards and development perspectives

This position paper seeks to reconcile the disparities of current SP programs described above. The observed similarities and widely-established structural features are adequately represented in the positions, without restricting the real-life heterogeneity. For this reason, many aspects are deliberately formulated in general, so that a clear direction is given but the actual implementation is reserved for the individual locations with their tradition and respective possibilities and limitations.

Since 2015 the Committee for Simulated Patients of the German Association for Medical Education (GMA) has developed the minimum standards and development perspectives in a multi-level consensual process in a total of seven workshops, on the basis of the current state of research, international recommendations and the status quo. The minimum standards include requirements that an SP program must meet in light of the current scientific discourse for simulated patients. The development perspective however additionally describes recommendations for the future development of SP programs. The individual standards and recommendations are thematically clustered (see attachment 1 ).

## 4. Conclusion

This position paper is the result of a multi-year process aimed at bringing together the current state of SP programs in German-speaking countries, national and international research perspectives and existing standardization and consensus processes. The paper’s intention is to explicitly describe existing structures and processes of SP programs and at the same time to shape the course for future developments, based on current research. 

Medical didactic and health and science policy changes such as the introduction of the new federal final examinations in Switzerland [26] or the German Federal Master Plan 2020 [27] with the planned practical exam formats (OSCEs) in the 2^nd^ and 3^rd^ state examinations ensure that the importance of SP programs is steadily increasing. In turn there is building pressure for the method to be as valid and reliable as possible, to be secure and dependable from a legal perspective and to work according to established, comprehensible and uniform standards. The position paper should help address these processes and the associated expectations and to anchor the didactic method of SPs with greater scientific foundation. In addition, this paper should also act as a contribution to the discussion of upcoming faculty development processes and didactic discourses.

As is usual with consensual processes, not all opinions are explicitly represented and sometimes contradictory positions have to be reconciled in compromise solutions. Nevertheless, key contributions to the discussion and opinions in recent years are presented and directions are pointed out in which the SP method can develop in German-speaking countries and, in the opinion of the authors and numerous participants involved in the creation process, should develop. Of course the wider discussion itself is not over. In conclusion there is the expectation and hope that the discussions about and research on the SP method will continue to be equally vivid and fruitful in the future and that the positions presented here will provide important stimuli for this. 

## Notes

The position paper was accepted by the GMA executive board at 03-06-2019.

^1^ The “Simulated Patients” committee of the Society for Medical Education (GMA) was renamed the “Simulated Persons” Committee in February 2019. This text uses the old name because the position paper was developed prior to the name change.

^2 ^ We would also like to thank all participants of the various workshops as well as the members of the Committee of Simulated Patients of the Society for Medical Education, who participated in the development of the positions and without whom the broad consensus and the position paper in its present form would not have been possible.

## Competing interests

The authors declare that they have no competing interests. 

## Supplementary Material

Minimum Standards and Development Perspectives

## Figures and Tables

**Table 1:**
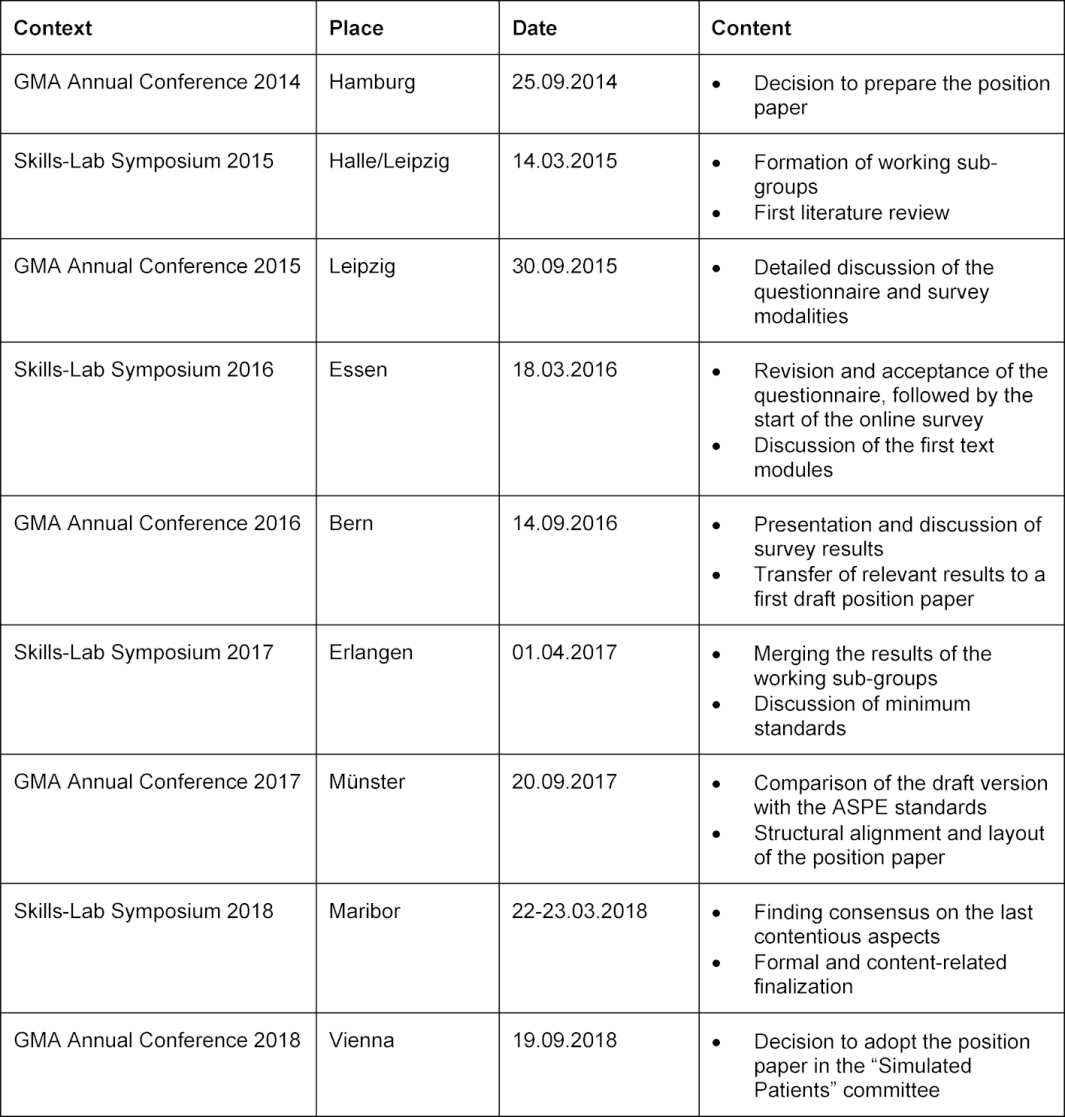
Timeline of the preparation process as well as content orientation of the workshops
