# Supplementary material for: Minimum standards and development perspectives for the use of simulated patients – a position paper of the committee for simulated patients of the German Association for Medical Education
Source: GMS J Med Educ. 2019 May 16;36(3):Doc31. doi: 10.3205/zma001239 (PMC6545616; doi:10.3205/zma001239)
Supplement: Minimum Standards and Development Perspectives [file JME-36-3-31-s-001.pdf]

<table>
<tr><th>Minimum standard</th><th>Development perspective</th></tr>
<tr><td colspan="2">1. Safe working environment</td></tr>
<tr><td colspan="2">1.1 Safe Working Practices</td></tr>
<tr><td>Protective measures for SPs are documented and there is a plan for how they will be implemented. The protective measures are presented transparently and the actual implementation is individually coordinated with the separate SPs.
The protective measures cover at least the following topics:
Limiting physical and mental stress,
taking into account the personal requirements of the individual SPs, including the individual endurance,
providing appropriate spatial requirements,
supporting SPs in case of unjustified complaints by students or lecturers.</td><td>The SPs are regularly involved in the development of the protective measures.
There is an institutionalized reporting system for newly identified risks (such as evaluations by SPs or regular supervision).</td></tr>
<tr><td colspan="2">1.2 Confidentiality</td></tr>
<tr><td>Applicable privacy policies must be respected at all times for and by anyone involved in the simulations.</td><td></td></tr>
<tr><td colspan="2">1.3 Respect</td></tr>
<tr><td>The right of self-determination of the SPs is respected. They receive sufficient information about all aspects of the job to make informed decisions.</td><td></td></tr>
<tr><td colspan="2">2. Case development</td></tr>
<tr><td colspan="2">2.1 Preparation of the case presentation</td></tr>
<tr><td>There are clear guidelines that a role script and its concept must meet:
Learning goals are clear and measurable (for example according to SMART criteria)
relevant subject content is authorized by experts</td><td></td></tr>
<tr><td colspan="2">2.2 Essential components of the case/role script</td></tr>
<tr><td>The case/script must contain at least:
All relevant information about the role (for example, situation and background story, biography, character and behavior, characters and symptoms to be simulated, assignments/keywords)
relevant props
relevant information on the SPs (such as age, body type, exclusion criteria)</td><td></td></tr>
</table>

Minimum standard	Development perspective
3. SP training	
**3.1 Training prerequisites, preparation**	
• All training is planned specifically.<br>• All training is tailored to the respective training purpose.	
**3.2 Training the role presentation**	
• Role training of the SPs takes place for each new role.<br>• Role training takes into account the respective goals, framework conditions and formats of the courses or exams.<br>• Training covers the essential aspects of the role to be presented and, where appropriate, aspects of standardization.	• Further training (for example, general role presentation skills or basics of communication) is offered.
**3.3 Feedback training**	
• SPs are properly trained in giving feedback, insofar as that is appropriate for their assignments.<br>• Training includes the goals, methods and framework conditions of the feedback.	
**3.4 Training on the use of assessment tools**	
• If the SPs use assessment tools to assess students' performance, they will receive training. The training aims to provide the SPs with the context and format of the assessment tool.<br>• The SPs are aligned according to their assessment behavior in order to achieve high inter-rater reliability.	
**3.5 Reflection on the training processes**	
• The coaches reflect on the training process and it is adapted if necessary.	• To evaluate the training process, the SPs are interviewed and, if necessary, standardized instruments are used.
4. SP program management	
**4.1 Objective**	
• There are written fixed objectives of the SP program.	• As far as possible, measurable sub-goals are identified and checked at fixed intervals.
**4.2 Expertise**	
• There is a permanent SP coach with clear responsibility for the SP program.<br>• The work with SPs is based on the current state of research.<br>• The team responsible for the SP program has the necessary skills (for example in the areas of theater education, human resources, organizational skills) and capacity to fully meet the minimum requirements set out in this position paper.	• All faculty members who regularly work with SPs receive adequate training from at least four teaching units following a fixed concept.

Minimum standard	Development perspective
**4.3 Policies and procedures**	
• In its communication, the SP program uses the nomenclature currently valid in German-speaking countries, based on the German-language handbook “Simulationspatienten” published in 2018 [29].<br>• Relevant framework conditions (for example, rights and obligations, remuneration, data protection, etc.) of SP activity are regulated in written contracts with the SPs. These should be approved by the legal department of the institution.	• Non-contractual aspects of cooperation are determined in writing for all participants (lecturers, SPs, SP coaches).<br>• These guidelines are available for all involved parties and are updated regularly.
**4.4 Recording and data management**	
• Archiving and access to SP case scenarios is organized according to a fixed system that takes copyright issues into account.<br>• Confidential data (video material, consent forms, personal SP data, etc.) are securely stored in accordance with the statutory data protection rules and destroyed on time.	
**4.5 Team management**	
• There is a permanent SP coach with clear responsibility for the SP program.<br>• There is a well-regulated selection process with clear selection criteria for including new SPs in the pool (such as memory, ability to reflect, reliability).<br>• The SPs used can credibly represent the requirements of the role (age, gender, appearance, etc.). Props (such as makeup, clothing, equipment) may be used.<br>• The assigned SPs can credibly represent the internal attitude and the emotions of the role.<br>• The assignments of SPs are appropriately remunerated. The lower limit does not fall below the statutory minimum wage applicable to employees.	• Responsibility for the SP program is enshrined in the employment contract and/or job description.<br>• The employment contract is open-ended.<br>• General, non-role-specific job descriptions/requirement profiles for the SPs are fixed in writing and published.<br>• If possible, several team members are included in the selection of SPs.<br>• There is a pool of props.<br>• The remuneration of the SPs is based on the level of comparable qualified freelance work.<br>• SPs are supported in (local as well as national or international) networking activities.<br>• The SP pool reflects the diversity of people to be represented in the simulation exercises.
**4.6 Quality management**	
• The SP program and its work are regularly evaluated with appropriate methods.<br>• The quality of the role presentation of each SP is checked regularly by the SP coach.<br>• The quality of the feedback of each SP is checked regularly by the SP coach.<br>• Evaluators and stakeholders are informed when changes to the program have been made as a result of their feedback.<br>• There is a regular subject-related exchange between teaching co-ordinators and staff of the SP program.<br>• There are measures to ensure the quality of the work of SP coaches. These include at least:<br>o Funded education and training,<br>o participation in SP-related committees and networks,<br>o support for an evidence-based development of the SP program.	• A wide selection of the people involved in the program (SP coaches, teaching staff, SPs, students, etc.) are interviewed intensively on quality at fixed intervals and then changes in the program are discussed and implemented.<br>• Measures to ensure the quality of work of the SP coaches also include the support of research activities.<br>• When reviewing the quality of role presentation, standardized instruments are used.<br>• When reviewing the quality of feedback, standardized tools are used.

Minimum standard	Development perspective
**4.7 Positioning of the program**	
• The SP program is visible to the outside world. It can be found on the Internet/Intranet using obvious keywords and locations. The page contains the name and contact details of a contact person as well as basic information about the SP program.<br>• All responsible teaching staff and faculty bodies are regularly informed about the simulated patient program.<br>• If faculty teaching courses with SPs are available, the students are regularly informed about these.<br>• If several SP programs exist at a faculty, they are networked with each other.<br>• Parallel programs work according to the same standards or make the differences clear.<br>• The working conditions of the SPs are designed so that there is no competition for the SPs between parallel programs.<br>• The SP program is an integral part of medical education.	• Existing media/communication channels to the general public are used.<br>• Any existing high-profile public events will be used to present the SP program.<br>• The SP program is shown in the organizational chart of the parent institution.<br>• If there are several SP programs in one site, organizational synergy effects are used (for example, personnel management, scheduling, billing).<br>• The financing of the SP program is secured in the long term.<br>• The SP program is anchored in the curriculum.
5. Professionalism	
**5.1 Career planning**	
• Those responsible for the SP program have the opportunity to acquire further qualifications.	• Research with and about SPs is made possible and institutionally funded.
**5.2 Scientific character**	
• Those responsible for SPs are familiar with a large number of fields of application and methods described in the literature for SPs.<br>• People responsible for SPs know the current scientific body of knowledge on the SP method and take this into account.<br>• People responsible for SPs actively participate in SP-related specialist associations and networks.	• SP-related research is taking place.<br>• Insights from own research with SPs are made accessible to a wide scientific audience (publications).<br>• Those responsible for SPs contribute to the advancement in the use of SPs (for example in the areas of best practices and evidence-basing).
**5.3 Leadership**	
• Those responsible in the SP program live up to their leadership function internally and their representational function to the outside world and continue to develop this (for example leadership training).	
